# Cardiotoxicity in Breast Cancer Therapy: Risks, Mechanisms, and Prevention Strategies

**DOI:** 10.3390/medsci13030130

**Published:** 2025-08-14

**Authors:** Annisa Eka Fitrianti, Nadea Olyvia Wardani, Astri Astuti, Kusnandar Anggadiredja, Lia Amalia, Risani Andalasia Putri, Zulfan Zazuli

**Affiliations:** 1 Master’s Program in Pharmacy, School of Pharmacy, Institut Teknologi Bandung, Bandung 40132, Indonesia; annisaekaf@gmail.com (A.E.F.); nadeaolyviaward@gmail.com (N.O.W.); 2 Department of Pharmacy, Dr. Hasan Sadikin General Hospital, Bandung 40161, Indonesia; 3 Department of Cardiology, Dr. Hasan Sadikin General Hospital, Bandung 40161, Indonesia; astriastuti85@gmail.com; 4 Department of Pharmacology—Clinical Pharmacy, Institut Teknologi Bandung, Bandung 40132, Indonesia; kusnandar_a@itb.ac.id (K.A.); lia_amalia@itb.ac.id (L.A.); 5 Department of Pharmacy, Dharmais Cancer Hospital, Jakarta 11420, Indonesia; risani.andalasia@dharmais.co.id

**Keywords:** breast cancer, anthracyclines, trastuzumab, cardiotoxicity, cardioprotective

## Abstract

Breast cancer is the most prevalent cancer in women. Anthracyclines are commonly used as the first line of treatment, often combined with other agents, including trastuzumab. Despite their efficacy, both drugs pose a risk of cardiotoxicity, which may impair patients’ quality of life (QoL) and hinder treatment persistence. Anthracycline-induced cardiotoxicity is dose-dependent and generally irreversible, whereas trastuzumab is associated with potentially reversible cardiac dysfunction. This review discusses the risk factors and biological mechanisms underlying chemotherapy-induced cardiotoxicity in breast cancer and explores effective strategies for prevention and treatment. It has been demonstrated that several cardioprotective strategies, such as treatments with angiotensin-converting enzyme inhibitors (ACEis), angiotensin receptor blockers (ARBs), beta-blockers, and dexrazoxane, can help lessen cardiotoxic effects. A better understanding of cardioprotective strategies may help optimize cancer treatment without compromising cardiovascular function.

## 1. Introduction

Breast cancer arises from the uncontrolled proliferation of epithelial cells lining the ducts or lobules of the breast, making it the most common cancer among women, accounting for approximately one-third of all cancer diagnoses in this population [1]. According to Global Burden of Cancer Study (Globocan) data for 2022, the incidence of new breast cancer cases was 66,271, constituting 16.2% of the total 408,661 new cancer cases in Indonesia, with a prevalence of 209,748 cases (20.6%) during the past five years [2].

For many years, anthracycline-based chemotherapy, including doxorubicin, has been the primary option for breast cancer treatment, frequently in conjunction with targeted therapy like trastuzumab [3,4]. However, its use is associated with toxicities ranging from mild to severe, with both short- and long-term implications [5]. Cardiotoxicity, marked by a reduction in the left ventricular ejection fraction (LVEF), is a prevalent adverse effect. According to Wang et al. patients with a cardiovascular risk may experience more severe effects from cardiotoxicity brought on by doxorubicin and trastuzumab, which may ultimately make it more difficult to continue treatment and raise the risk of death [6]. Cardiotoxicity associated with trastuzumab frequently manifests in the initial phases of treatment and typically presents without clinical symptoms [7].

This article aims to review cardiotoxicity as a significant consequence of breast cancer treatment, particularly associated with doxorubicin and trastuzumab. In conjunction with advancements in cancer treatment, the side effects manifesting as a compromised cardiac function need to be taken into consideration as they can disrupt the continuity of therapy and adversely affect the patient’s quality of life (QoL). This article is aimed at explaining the fundamental biological mechanisms and identifying risk factors associated with cardiotoxicity. This article also reviews various strategies for the prevention and therapy of cardiotoxicity to mitigate its adverse effects on cardiac function.

## 2. Literature Searching

This review summarizes the results of several studies related to cardiotoxicity in breast cancer therapy, particularly anthracycline-based chemotherapy and trastuzumab. The included studies were identified through the PubMed database using the keywords “cardiotoxicity,” “doxorubicin,” “trastuzumab,” “breast cancer,” and “left ventricular ejection fraction.” An initial total of 402 articles published within the past 10 years were retrieved. The search results were further refined by limiting them to studies involving human subjects, yielding 185 articles. Studies that did not specifically examine cardiotoxicity related to anthracyclines and trastuzumab were excluded. Additionally, research communications, theses, review articles, expert opinions, and irrelevant studies were also excluded. Following abstract screening based on predefined inclusion criteria, 26 studies were ultimately selected for this review. Most of these studies explored the comparative cardiotoxic effects of different treatment regimens, identified risk factors associated with cardiotoxicity, and evaluated various cardioprotective strategies. Differences in findings between studies were also discussed.

## 3. Breast Cancer

Breast cancer is a disease that arises from the glands, ducts, and supportive tissues within the breast. This disease is among the most prevalent forms of cancer in women, alongside cervical cancer. Cancer cells proliferate uncontrollably and may develop in the lobules (milk-producing glands), ducts (milk-transporting channels), adipose tissue, or connective tissue within the breast [8]. The initial proliferation of cancer cells often transpires within the ducts and/or lobules (in situ), which at this juncture is not life-threatening; nonetheless, cancer cells may disseminate to adjacent breast tissue (invasively), resulting in the formation of tumors that manifest as lumps or thickening. Invasive breast cancer can metastasize to adjacent lymph nodes or other organs [9].

According to the 2022 Globocan statistical data from the World Health Organization (WHO), breast cancer has the second highest incidence following lung cancer. Worldwide, there are 2,296,840 newly diagnosed cases of breast cancer, resulting in a mortality rate of 666,103, positioning it as the fourth greatest cause of cancer-related fatalities. As of the end of 2022, a total of 8,178,393 individuals were diagnosed with breast cancer within the preceding five years [2].

In Asia, 48 countries exhibit diverse trends in the breast cancer incidence throughout time. From 1990 to 2017, the incidence of breast cancer rose markedly, with the age-standardized incidence rate (ASIR) increasing by 54% in Central Asia, 89.5% in East Asia, and 76.7% in South Asia [10]. In 2018, breast cancer represented 10% of all cancer cases in Asia, totaling 845,400 cases [11]. By 2040, breast cancer diagnoses are projected to reach 1.34 million [2].

Genetic mutations mostly cause breast cancer, particularly those affecting tumor suppressor genes such as BRCA-1, BRCA-2, p53, and PTEN [1]. Mutations in the BRCA1 and BRCA2 genes, which are crucial to DNA repair and cellular growth regulation, can elevate the risk of tumorigenesis. These two genes experience heritable functional alterations that influence the expression profile of oncogenes and contribute to malignancy [12,13].

The predominant and generally accepted classification of breast cancer relies on an immunohistochemical methodology. This methodology delineates critical biomarkers, including the estrogen receptor (ER), progesterone receptor (PR), and human epidermal growth factor receptor 2 (HER2), utilized to categorize breast cancer into five subtypes: luminal A, luminal B, HER2 positive, triple-negative breast cancer (TNBC), and normal-like [14,15]. The TNBC subtype is defined by the lack of the expression of all three receptors, resulting in distinct biological features compared to other subtypes [14].

The HER2-positive subtype of breast cancer is defined by the overexpression or amplification of the HER2 protein, representing roughly 20–30% of all breast cancer [16,17]. Alongside the TNBC subtype, HER2-positive breast cancer demonstrates a more aggressive behavior and has a diminished overall prognosis relative to other types [18,19]. The National Cancer Institute study on survival rates indicates that women with the luminal A subtype have the most favorable prognosis, with a 5-year relative survival rate of 94.4%. The luminal B subtype has a survival rate of 90.7%, followed by the HER2 subtype at 84.8%, whilst the TNBC subtype exhibits the lowest survival rate at 77.1% [20].

## 4. Treatment of Breast Cancer

Over the past twenty years, the management of early breast cancer (stage I–IIIA) has advanced considerably, incorporating a combination of pharmacological agents, radiotherapy, and surgical interventions. Nonetheless, anthracyclines continue to be a principal element of breast cancer treatment, sometimes utilized with novel targeted therapies such as trastuzumab [4]. A meta-analysis by Bradley et al. indicates that anthracycline-based regimens, particularly when combined with taxanes, can decrease the chance of breast cancer recurrence within 10 years by as much as one-third compared to non-chemotherapy approaches. This medication also decreases the death rate from breast cancer by 20–25% [21].

Doxorubicin is a cytotoxic drug belonging to the anthracycline class, developed from Streptomyces species. The mechanism of action of anthracyclines is essentially analogous, specifically by intercalating DNA bases into the molecule, hence perturbing the DNA helical structure. This disruption impedes DNA synthesis and influences the activity of the DNA topoisomerase II enzyme. Furthermore, anthracyclines can generate iron-mediated free radicals that may target and harm rapidly proliferating cells [22,23,24]. Trastuzumab is a targeted therapeutic drug beneficial in treating HER2-positive breast cancer. Trastuzumab is a humanized recombinant monoclonal antibody specifically engineered to bind the extracellular domain of the HER2 protein, thereby inhibiting dimerization and obstructing downstream intracellular signaling pathways, which leads to cell cycle arrest and the inhibition of DNA repair following chemotherapy [25,26,27,28].

During the 2005 annual meeting of the American Society of Clinical Oncology (ASCO), the advantages of trastuzumab in conjunction with anthracycline/taxane-based adjuvant therapy for HER2-positive breast cancer were presented for the first time. The meta-analysis results indicate that one year of trastuzumab therapy in early-stage HER2-positive breast cancer markedly enhances the disease-free survival (DFS) and overall survival (OS), while diminishing the risk of recurrence and mortality [21]. Consistent with prior research findings, the amalgamation of trastuzumab with chemotherapy has demonstrated a substantial enhancement in DFS by 36–58% and OS by 24–59% [29,30,31,32].

The Neoadjuvant Herceptin (NOAH) clinical trial was performed to evaluate the efficacy of treatment in HER2-positive breast cancer patients, with 118 patients receiving chemotherapy only. In contrast, 117 more patients underwent a regimen of chemotherapy combined with trastuzumab. The trial results indicated that the 5-year event-free survival (EFS) was 58% (95% CI 48–66) for the group receiving chemotherapy with trastuzumab, in contrast to 43% (95% CI 34–52) for the group receiving chemotherapy alone [33]. A further study has shown that the use of adjuvant trastuzumab alongside paclitaxel can diminish the risk of local–regional recurrence and mitigate toxicity in HER2-positive breast cancer patients with tumors ≤ 2 cm and no lymph node involvement [34].

Other treatment types in early-stage breast cancer include the use of systemic adjuvant therapy to reduce the risk of recurrence. Treatment decisions are based on an individual’s risk of relapse and the likelihood of a response, taking into account factors such as the ER/PR and HER2 status [35]. For patients with hormone receptor-positive and HER2-negative breast cancer, endocrine therapy with tamoxifen or aromatase inhibitors remains the cornerstone of adjuvant treatment [36]. In cases of HR+/HER2− breast cancer with a high risk of recurrence, CDK4/6 inhibitors such as abemaciclib have been shown to provide significant benefits in the adjuvant setting when combined with endocrine therapy [37,38,39]. The combination of abemaciclib and endocrine therapy significantly improved the invasive disease-free survival (IDFS) compared to endocrine therapy alone (HR 0.75; 95% CI: 0.60–0.93), with a two-year IDFS rate of 92.2% in the combination group versus 88.7% in the endocrine therapy-only group [39].

In addition, for patients with germline BRCA1/2 mutations and HER2-negative status, adjuvant olaparib has been shown to significantly reduce the risk of recurrence. A study with a median follow-up of 2.5 years reported a three-year IDFS rate of 85.9% in the olaparib group, compared to 77.1% in the placebo group [40]. For patients whose breast cancer progresses after the first-line treatment with trastuzumab or pertuzumab, the standard second-line therapy is an antibody–drug conjugate such as trastuzumab emtansine (T-DM1). Treatment with trastuzumab emtansine was associated with a median progression-free survival of 9.6 months and a median overall survival of 30.9 months [41]. Furthermore, immunotherapy—particularly immune checkpoint inhibitors such as pembrolizumab and atezolizumab—has shown benefits in patients with TNBC, especially those who are PD-L1 positive. The KEYNOTE-522 study reported improvements in the pathologic complete response (pCR) and EFS in early-stage TNBC patients receiving neoadjuvant pembrolizumab [42].

## 5. Cardiotoxicity

The National Cancer Institute characterizes cardiotoxicity as a toxic illness impacting the heart. This concept encompasses both the direct impact of the drug on cardiac function and the indirect effects resulting from alterations in the hemodynamic flow or thrombotic occurrences [43]. The Cardiac Review and Evaluation Committee defines cardiotoxicity as a condition encountered by patients following an anticancer treatment, characterized by one or more of the subsequent criteria:

1. Cardiomyopathy is defined by a reduction in the LVEF or a decrease in ventricular septal motion.
2. The manifestation of heart failure symptoms.
3. Tachycardia.
4. A reduction in the LVEF of ≥5% to <55% is associated with heart failure symptoms, or a decline in the LVEF of ≥10% to <55% in the absence of symptoms [44].

However, despite efforts to standardize criteria, there remains considerable heterogeneity in definitions of cardiotoxicity, especially in relation to LVEF cutoffs. Different organizations propose varying thresholds. The American Society of Echocardiography (ASE) and the European Society of Cardiovascular Imaging (EACVI) guidelines indicate that cardiotoxicity is diagnosed when there is a reduction in the ejection fraction (EF) of ≥10%, with an absolute value of <53% [45].

The European Society of Cardiology (ESC) establishes that the classification is divided into two main categories: Symptomatic Cancer Therapy-Related Cardiovascular Dysfunction (CTRCD) and Asymptomatic CTRCD. Symptomatic CTRCD applies when a patient is experiencing symptoms of heart failure. The severity is graded as follows:

1. Very severe, HF requiring inotropic support, mechanical circulatory support, or the consideration of transplantation.
2. Severe, HF hospitalization.
3. Moderate, need for the outpatient intensification of diuretic and HF therapy.
4. Mild, mild HF symptoms, no intensification of therapy required.

Cancer therapy-related cardiovascular toxicity definitions for asymptomatic CTRCD:

1. Severe, a new LVEF reduction to,40%
2. Moderate, a new LVEF reduction by ≥10 percentage points to an LVEF of 40–49% or a new LVEF reduction by 10 percentage points to an LVEF of 40–49% and either a new relative decline in GLS by >15% from baseline OR a new rise in cardiac biomarkers.
3. Mild, an LVEF ≥ 50% and a new relative decline in GLS by >15% from baseline and/or a new rise in cardiac biomarkers [46].

A reduction in the LVEF can be classified as symptomatic or asymptomatic according to its degree of reversibility, as outlined by Plana et al.:

1. If the LVEF improves by 5% points from the baseline, the condition is considered reversible.
2. If the improvement reaches ≥10% points from the lowest point but is still more than 5% below the baseline, it is classified as partially reversible.
3. If the improvement is less than 10% points from the lowest point and is still more than 5% points below the baseline, the decrease in the LVEF is considered irreversible [47].

Cardiotoxicity can develop over a long period, starting from the beginning of treatment to at least four years after chemotherapy ends [48]. This condition can appear in several forms:

1. Acute or subacute, which occurs from the start of treatment until two weeks after the therapy is complete.
2. Chronic, which develops at least one year after the end of the therapy. Chronic cardiotoxicity is further classified into two types, namely early chronic if it appears in the first year after therapy and advanced chronic if it occurs in the years following the completion of the treatment [49,50].

This lack of uniformity complicates cross-trial comparisons and clinical decision-making. The variability may also impact the patient stratification, treatment discontinuation decisions, and eligibility for cardioprotective interventions.

In addition to the LVEF, newer modalities such as global longitudinal strain (GLS) are sensitive imaging biomarkers used to detect early subclinical changes in left ventricular contractility in patients undergoing chemotherapy with a cardiotoxic potential. A relative reduction in GLS exceeding 15% from the baseline has been reported as an accurate predictor of a subsequent decrease in the LVEF [45,47,51]. This condition is considered abnormal and is an early indicator of subclinical left ventricular dysfunction. However, to date, the use of GLS and its threshold in predicting cardiotoxicity has not been standardized [52].

Troponin I have also emerged as a biomarker for early myocardial injury. Several studies have shown that troponin I measurements after the start of chemotherapy are useful in predicting the incidence and severity of cardiotoxicity, especially in patients receiving anthracyclines and those undergoing combination therapy including trastuzumab [53,54,55]. Cardiac troponin, a regulatory protein in cardiomyocytes, is released into the circulation upon a myocardial injury. Studies indicate that troponin may detect cardiotoxicity at the preclinical stage, even before reductions in the LVEF are evident, especially in patients undergoing therapy with anticancer drugs [54,55,56]. Ultimately, the diverse definitions and evolving diagnostic modalities highlight the urgent need for unified, guideline-driven criteria for diagnosing cardiotoxicity. This would enhance consistency in clinical practice, enable better risk stratification, and facilitate more accurate inter-study comparisons.

## 6. Treatment and Cardiotoxicity

Among antineoplastic agents, drugs that are most at risk of causing cardiotoxicity can be categorized into two types:

1. Type I works through mechanisms such as anthracyclines. Cardiotoxicity in this type is dose-dependent and causes irreversible heart damage.
2. Type II, which works through mechanisms such as trastuzumab. In this type, cardiotoxicity is reversible, so the treatment can be temporarily stopped until the patient recovers and resumed if necessary. This is possible because there is no change in the ultrastructure of the myocytes, so the heart damage is not permanent [57,58].

Anthracyclines are a group of chemotherapy agents that are best known to have the potential to cause cardiac dysfunction, with a risk five times higher than non-anthracycline agents [59]. The risk of heart failure from the use of anthracyclines increases with increasing cumulative doses. At a dose of 400 mg/m^2^, the risk reaches 3–5%, while at a dose of 700 mg/m^2^, the risk can increase to 18–48% [60]. A 2013 meta-analysis reported that after a 9-year follow-up period, clinical cardiac toxic effects occurred in 6% of patients receiving treatment with anthracyclines, while subclinical cardiac toxic effects were found in 18% of patients [61]. Although doxorubicin doses exceeding 500 mg/m^2^ were frequently used in the past [62], current guidelines have limited the maximum cumulative dose to 450 mg/m^2^ [63]. Despite the ongoing debate regarding the long-term impact of various dosing regimens—largely due to differences in follow-up durations—the recommended lifetime cumulative dose of doxorubicin is now set at less than 450 mg/m^2^ to minimize the risk of cardiotoxicity [64].

Trastuzumab is an effective therapy for breast cancer, but its use can cause serious side effects in the form of significant cardiotoxicity. The HER2 receptor, which is the main target of trastuzumab, is not only found in tumor cells but is also expressed in cardiomyocytes. This receptor is thought to have a cardioprotective role in responding to cellular stress. Therefore, the use of trastuzumab can trigger a significant decrease in the LVEF and potentially cause heart failure [65,66]. This risk increases when trastuzumab is combined with other chemotherapies known to be cardiotoxic, such as anthracyclines [65]. A multicenter phase III trial conducted showed that the combination of trastuzumab with anthracyclines significantly increased the risk of cardiac dysfunction and heart failure. The results of this study revealed that up to 27% of HER2-positive metastatic breast cancer patients who received the combination experienced cardiotoxicity, compared to 8% in the group that only received anthracyclines. Other studies have also revealed the same thing, that the combination of anthracyclines and trastuzumab, with a higher percentage of doxorubicin compared to other types of anthracyclines, will further decrease the LVEF value when compared to anthracyclines alone [67].

A meta-analysis involving 21 prognostic studies examined patients undergoing treatment with anthracyclines, both with and without trastuzumab. The study identified that an absolute change in GLS of 2–3% and a relative change of 10–15% can be used as a threshold for detecting subclinical cardiotoxicity [68]. Similar findings were reported in a recent meta-analysis, which revealed that patients with cardiotoxicity experienced a 14.13% greater decrease in GLS than those who did not experience the condition. This further reinforces that GLS has a high predictive value in detecting cardiotoxicity and subclinical left ventricular dysfunction [69].

Another study found that a significant increase in troponin I (>30 ng/mL) in HER2-positive breast cancer patients undergoing anthracycline therapy followed by trastuzumab may be an early indicator of cardiotoxicity [56]. The ACC/AHA guidelines provide a Class I recommendation for measuring cardiac troponin (cTn) levels in patients presenting with chest pain and advise the use of high-sensitivity cardiac troponin (hs-cTn) assays for serial measurements [70]. This recommendation is supported by a subsequent study involving 533 breast cancer patients who underwent the serial monitoring of hs-cTnI and hs-cTnT levels during trastuzumab treatment. Patients with elevated baseline troponin levels (>40 ng/L for hs-cTnI and >14 ng/L for hs-cTnT) had a fourfold higher risk of developing left ventricular dysfunction [71].

Cardiotoxicity is a serious complication of both agents because it can interfere with the QoL and overall survival of breast cancer patients. Therefore, this aspect is a major concern in patient management [72,73]. An LVEF evaluation is recommended before and after anthracycline therapy, as well as before and every three months during trastuzumab use [74]. If the LVEF decreases to below 50%, with or without symptoms, the discontinuation of trastuzumab is recommended [17]. The cardiotoxic effects of the treatment force the cessation of therapy, which ultimately risks reducing the overall patient survival [75].

Cardiotoxicity is often the reason for the discontinuation of treatment, which has an impact on the increased risk of cancer recurrence and mortality. It is reported that this risk can reach up to 40% in the five years after undergoing chemotherapy [76]. The results of a randomized trial of adjuvant trastuzumab in combination with an anthracycline–taxane-based regimen showed a 12–15% decrease in the asymptomatic LVEF. However, few patients experienced the permanent discontinuation of treatment [77,78,79,80]. Approximately 18% of all patients experience trastuzumab-induced cardiotoxicity (trastuzumab-induced cardiotoxicity/TIC), which results in a delay in therapy. In line with previous research, TIC is the main cause of the discontinuation of adjuvant trastuzumab treatments, with rates reaching 62% [81,82,83].

Other research identifies the discontinuation of trastuzumab therapy as a significant prognostic factor, with patients who discontinue therapy having up to three times worse DFS and OS than those who complete treatment for one year or experience only a delay of less than 14 days [75]. In addition, Gong et al. reported that patients who discontinue adjuvant trastuzumab have a higher risk of disease recurrence and emphasized that the early discontinuation of therapy is a stronger prognostic factor for a poorer survival than cardiotoxicity itself [84].

## 7. Cardiotoxicity Mechanism

**Figure 1:**
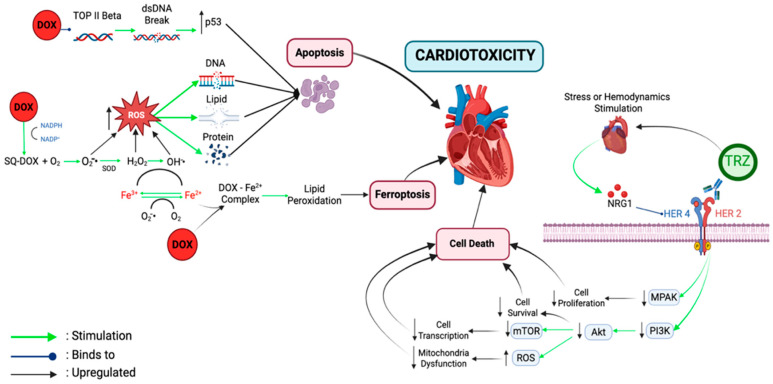
Doxorubicin-induced cardiotoxicity. In cardiomyocytes, DOX intercalates into DNA and inhibits topoisomerase II beta (TOP II beta), resulting in double-strand DNA (dsDNA) breaks and the activation of the tumor suppressor protein p53, which leads to apoptosis. Additionally, DOX undergoes a one-electron reduction by mitochondrial enzymes such as NADPH cytochrome P450 reductase, forming an unstable semiquinone radical. This semiquinone doxorubicin (SQ-DOX) undergoes redox cycling, generating superoxide anions (O_2_•^−^), which are subsequently converted into hydrogen peroxide (H_2_O_2_) either spontaneously or via superoxide dismutase (SOD). In the presence of free iron (Fe^2+^), H_2_O_2_ participates in Fenton reactions, producing highly reactive hydroxyl radicals (OH•). The accumulation of reactive oxygen species (ROS) causes significant oxidative damage to lipids, proteins, and DNA, particularly within the mitochondria. Furthermore, Fe^2+^ may form DOX–Fe complexes that amplify ROS production and promote the lipid peroxidation of mitochondrial membranes, contributing to mitochondrial dysfunction and triggering ferroptosis—a regulated, iron-dependent form of cell death. Trastuzumab-induced cardiotoxicity. Trastuzumab (TRZ), a monoclonal antibody targeting the extracellular domain of HER2, induces cardiotoxicity by disrupting the physiological HER2 signaling in cardiomyocytes. Under stress or hemodynamic stimulation, cardiac microvascular endothelial cells release neuregulin-1 (NRG1), which binds to HER4 on cardiomyocytes, facilitating HER4 homodimerization or HER4/HER2 heterodimerization. These receptor complexes activate downstream survival signaling pathways, particularly PI3K–Akt and MAPK, which play crucial roles in cell proliferation, mitochondrial integrity, and the protection against oxidative stress. Trastuzumab blocks HER2, thereby inhibiting HER4/HER2 heterodimerization and attenuating NRG1-mediated signaling. Consequently, the PI3K–Akt pathway is suppressed, leading to decreased nitric oxide (NO) production, impaired mitochondrial function, increased ROS accumulation, and ultimately cardiomyocyte apoptosis. The MAPK pathway, which also contributes to mitochondrial stability, is similarly disrupted.

The cardiotoxicity mechanisms of doxorubicin and trastuzumab involve different pathways, yet both may contribute to the development of myocardial dysfunction (Figure 1). The cardiotoxic effects of doxorubicin primarily arise from three interconnected mechanisms: oxidative stress, the dysregulation of cell death pathways, and epigenetic modifications [47,85,86]. In terms of oxidative stress, doxorubicin plays a role in producing reactive oxygen species (ROS) [87,88,89], as well as increasing iron mobilization [87,90]. When ferric ions bind anthracyclines, ROS are generated, promoting the redox cycling of ferrous to ferric ions. This redox cycle induces widespread cellular damage, affecting the mitochondrial membrane, nucleus, plasma membrane, and endoplasmic reticulum. As a result, there is a decrease in intracellular calcium levels, which has an impact on the decreased heart contractility [85,91,92].

Additional studies have shown that doxorubicin induces a cardiomyocyte injury through interactions with DNA topoisomerase II beta (TOP2B) [83]. Cardiomyocyte damage is not only influenced by ROS but also by the TOP2B–ROS–DNA complex. This complex causes the suppression of transcription factors (through p53 activation), DNA degradation, and apoptosis that cannot be avoided due to mitochondrial dysfunction [89].

Doxorubicin induces cardiomyocyte death via multiple pathways, including intrinsic and extrinsic apoptosis which occurs through both intrinsic and extrinsic pathways. Recent studies have also revealed that pyroptosis and ferroptosis contribute to the loss of functioning myocytes, especially through the upregulation of non-coding RNA that plays a role in terminal differentiation. These processes result in iron dysregulation and elevated lipid peroxidation, thereby exacerbating cardiomyocyte damage [85,87,90].

Doxorubicin causes an increase in the levels of labile iron in cells, which contributes to its cytotoxic effect. In another mechanism, doxorubicin and its metabolites can disrupt the balance of iron homeostasis by interfering with the expression of genes that play a role in iron metabolism. Furthermore, doxorubicin also inhibits the glutathione peroxidase activity in the cytosol and mitochondria, which triggers lipid peroxidation and ultimately causes ferroptosis [93]. In addition, doxorubicin can damage the cardiomyocyte mitochondria, which then causes heart failure through the induction of pathological autophagy. This induction has the potential to cause cell death rather than functioning as a normal mechanism for recycling and cleaning up damaged cellular components [85,94].

Doxorubicin-associated cardiotoxicity is also mediated through epigenetic modifications. This drug is known to downregulate DNA methylation, which has an impact on changes in the mitochondrial gene expression [95]. In addition, anthracyclines also increase histone deacetylation activity, which causes the deacetylation of certain proteins, such as α-tubulin [96]. Doxorubicin also affects the regulation of various microRNAs, although the further impact of these changes is not yet fully understood [97].

The precise mechanism of trastuzumab-induced cardiotoxicity remains incompletely understood. In vivo investigations have shown that trastuzumab causes ultrastructural changes in rat heart tissue and significantly alters the expression of genes involved in the cardiac function, mitochondria, and DNA repair [98].

The inhibition of the NRG1/HER signaling pathway and its downstream pathways has long been considered the main mechanism contributing to TIC. In addition, recent research highlights the role of autophagy inhibition and changes in cellular metabolic pathways in cardiomyocytes as potential factors in the development of cardiotoxicity [99,100,101]. Mohan et al. found that treatment with trastuzumab led to the decreased expression of proteins involved in the autophagy signaling pathway, including ATG5-12, ATG7, ATG14, and Beclin. In addition, their research shows that trastuzumab-induced autophagy inhibition contributes to increased ROS production in cardiomyocytes, potentially leading to oxidative stress and cellular damage [101].

HER2 is not only expressed in tumor tissue but is also found in adult cardiomyocytes along with other family members, such as HER1, HER3, and HER4 [102], although to a lesser extent than in the embryonic heart [103]. Together with its ligand, NRG1, HER2 plays an important role in maintaining the function and survival of cardiomyocytes. When the heart experiences hemodynamic stress, the microvascular endothelial cells release NRG1 [103,104,105], which then binds to HER4 and triggers HER4/HER4 homodimerization or HER4/HER2 heterodimerization, activating downstream signaling pathways such as mitogen-activated protein kinase (MAPK) and phosphoinositide 3-kinases (PI3K)-Akt to support heart survival and function [25,106].

This study demonstrates that the HER2 deletion in the murine model causes spontaneous dilated cardiomyopathy and increases the susceptibility to cardiovascular stress. Collectively, these findings underscore the critical role of HER2 in preserving the cardiac structure and function, particularly under stress conditions, especially under conditions of physiological or pharmacological stress, which explains how the HER2 inhibition by trastuzumab can contribute to cardiotoxicity [107,108].

Akt plays an important role in cell survival by activating various proteins through phosphorylation, which ultimately prevents apoptosis and supports cell growth [109]. Research conducted by Ravingerova et al., using a mouse model with chronic cardiac ischemia, showed that the Akt activation increases the glucose and lipid metabolism in cardiomyocytes, allowing for better nutrient absorption and maintaining energy during hypoxic conditions [110]. In addition, the PI3K-Akt pathway also contributes to cell protection by stimulating the production of NO in adult ventricular myocytes, which helps fight oxidative stress. Akt also plays a role in regulating mitochondrial respiration, thus suppressing ROS production and increasing cell survival [111]. However, when HER2 is inhibited, as in the treatment with trastuzumab, the PI3K-Akt pathway cannot be activated, causing an increase in ROS accumulation in cardiomyocytes and ultimately triggering apoptosis and cardiac dysfunction [111,112].

The MAPK (MEK/ERK) pathway also plays a role in the survival of cardiomyocytes by amplifying external signals that support cell proliferation and differentiation [113,114]. The decrease in HER2 activity due to trastuzumab weakens the MEK/ERK pathway, which then triggers apoptosis [115]. The inhibition of this pathway also increases the number of mitochondrial permeability transition pores (mPTPs), causing an increased sensitivity to excess calcium (Ca^2+^), the excessive production of cytotoxic ROS, and a decreased gap junction permeability, which ultimately contributes to cardiomyocyte damage [116].

## 8. Risk Factors of Cardiotoxicity

Key risk factors for chemotherapy-induced heart failure include the cumulative drug dose, an age over 65, concurrent treatment with or prior to thoracic radiation, the co-administration of cardiotoxic agents, and pre-existing cardiovascular disease [117]. Some known risk factors associated with the development of anticancer treatment-induced cardiotoxicity are described below:

1. Age

An advancing age significantly elevates the risk of cardiotoxicity. The results showed that women aged 65 years and over who received a combination of anthracyclines and trastuzumab, as well as women aged 75 years and over who received trastuzumab alone, experienced an increased risk of heart failure [65]. In line with other studies reporting a consistently high risk in women aged 65 and over, both those receiving anthracyclines [118] and combinations of anthracyclines with trastuzumab or trastuzumab alone had higher risks compared to those receiving other types of chemotherapy [119].

2. Hypertension

Hypertension is a prominent risk factor for doxorubicin-induced cardiotoxicity. This is most likely due to the pressure and initial damage to the heart before treatment, which then increases the risk of further damage [120]. Advances in the monoclonal antibody, tyrosine kinase, and other molecular target-based cancer therapies have significantly improved the survival of patients with advanced cancer, but these innovations also raise concerns about secondary hypertension, which can contribute to left ventricular dysfunction and be a major factor in adverse cardiotoxicity [121].

3. Overweight and Obesity

Epidemiological studies show that being overweight or obese not only increases the risk of breast cancer but also reduces the effectiveness of the treatment, lowers the QoL after diagnosis, and increases cancer mortality [122,123]. Obesity markedly increases the risk of cardiotoxicity, especially when doxorubicin is used alone or in combination with trastuzumab. Individuals who are obese or overweight have a higher risk of cardiotoxicity after an anthracycline-based therapy [124,125].

4. Diabetes

Diabetes is independently associated with an elevated risk of anticancer-induced heart failure. Several mechanisms that play a role in increasing this risk include lipotoxicity, hyperglycemia, and mitochondrial dysfunction, which contribute to the development of cardiotoxicity [126].

5. Dyslipidemia

Hyperlipidemia has been investigated in the context of other cardiovascular risk factors, such as hypertension and diabetes. Research shows that patients with these conditions have a higher risk of developing heart failure due to anthracyclines than those who do not have these risk factors. Therefore, dyslipidemia has the potential to worsen anthracycline-induced cardiotoxicity [127].

The ESC recommendations support the utilization of the newly established Heart Failure Association (HFA) and International Cardio-Oncology Society (ICOS) baseline risk stratification score [128]. The HFA-ICOS score was formulated following a systematic review of the literature concerning seven categories of potentially cardiotoxic cancer treatments, including anthracycline chemotherapy and HER2-targeted therapies [46].

The likelihood of developing cardiovascular toxicity associated with cancer treatment (CRT-CVT) in patients who have had a potentially cardiotoxic therapy is directly proportional to the presence of four types of risk factor categories [128].

1. Baseline risk evaluated using the HFA-ICOS risk assessment instruments.

This risk evaluation is predicated on the cardiovascular risk factors, prior cardiovascular disease history, cancer history, and previous cancer therapy. This evaluation entails a clinical examination, an electrocardiogram (ECG), and, in instances of a pre-existing cardiovascular disease or cancer therapies associated with a heightened risk of CTR-CVT, the measurement of the cTn, natriuretic peptides (NPs), LVEF, and GLS via echocardiography.

2. The type and dosage of the oncological treatment

Each chemotherapy agent may present a distinct risk of CTR-CVT, ranging from a negligible to a significantly elevated risk, typically associated with the administered doses, including both individual and cumulative doses (e.g., cumulative doses of anthracyclines), as well as the concurrent or sequential administration of additional oncological therapies (e.g., anthracyclines combined with trastuzumab and radiotherapy).

3. CRT-CVT occurring with the delivery of treatment

This encompasses nearly all types of cardiovascular injury across various cardiovascular levels, possessing diverse pathophysiological significance and therapeutic relevance.

4. The evaluation of clinical conditions, echocardiography, and biomarker assessments

This reappraisal should occur in all clinical settings at 12 months and at 3 and 12 months for patients initially classified as high- and very-high risk. The echocardiogram must encompass a comprehensive assessment of the left and right ventricular function and, when feasible, the evaluation of the global longitudinal strain in comparison to baseline data. The clinical assessment must encompass cardiovascular risk factors in accordance with the ESC Guidelines on Prevention. This evaluation should take place at 12 months (and at 3 months for participants previously recognized as high- and extremely-high-risk during therapy) [129].

## 9. Cardioprotective

As a follow-up, the ESMO released updated guidelines in 2020, which suggest that in patients with a mild asymptomatic LVEF decline, trastuzumab can be continued with an additional cardioprotective therapy. This condition is defined as a decrease in the LVEF of ≥10% from baseline or a decrease to a range of 40–<50% [130]. Cardioprotective therapies, including angiotensin-converting enzyme inhibitors (ACEis), angiotensin receptor blockers (ARBs), and beta-blockers, have been shown to prevent cardiac remodeling and reduce mortality in patients with cardiac dysfunction [131]. The current evidence suggests that this therapy plays an important role in preventing the discontinuation of adjuvant trastuzumab in HER2-positive breast cancer patients [132,133]. In addition, experimental animal studies [26], observational studies [134], and small-scale randomized trials report that this cardioprotective intervention can help maintain the left ventricular function in patients undergoing cardiotoxic chemotherapy [135,136,137,138,139].

A study demonstrated that the use of lisinopril and carvedilol significantly improved the cardiotoxicity-free survival compared to the placebo. In addition, the group receiving this therapy experienced fewer treatment discontinuations. In HER2-positive breast cancer patients undergoing anthracycline and trastuzumab therapy, the use of lisinopril or carvedilol as cardioprotective agents may help reduce the risk of the trastuzumab discontinuation due to cardiotoxicity [132]. Similar findings were reported by Cardinale et al., who demonstrated that enalapril effectively prevents anthracycline-induced cardiomyopathy [140]. Kaya et al. also reported similar benefits with carvedilol [137].

Several studies have also shown that the concurrent administration of candesartan is effective in preventing a decrease in the LVEF after chemotherapy [135,136,140]. The protective effect of candesartan against a decrease in the LVEF persists for up to one year after the completion of doxorubicin chemotherapy, while the protective effect of carvedilol begins to diminish after this period. This cardioprotective effect was observed even at a minimal dose of 4 mg per day. Candesartan demonstrated a lower incidence of early subclinical cardiotoxicity and a superior preservation of the LVEF compared to carvedilol and stronger protection against the LVEF decline. Notably, all patients in the candesartan group who developed early subclinical cardiotoxicity showed a complete recovery by the end of the study, with no significant difference in the LVEF between the beginning and end of the study [140].

The results of a study conducted by Pituskin et al. found that bisoprolol (beta-blockers) was more effective in reducing the decline in the LVEF and allowed more patients to continue trastuzumab therapy without interruption compared to perindopril (ACEi) and the placebo [141]. In contrast, another study conducted by Gulati et al. reported that candesartan (ARB) has a protective effect against the decreased LVEF, while metoprolol (beta-blockers) and the placebo did not show a significant impact on patients receiving epirubicin and trastuzumab [136].

Although the specific agent selection among beta-blockers and ACEis remains debated, several studies show that carvedilol and nebivolol have the best cardioprotective effect on patients undergoing anthracycline therapy [137,142,143]. Current evidence supports the safety and efficacy of neurohormonal agents, including Renin–Angiotensin–Aldosterone System (RAAS) inhibitors and beta-blockers, in mitigating cardiotoxicity. In addition, several studies have also reported a small improvement in the left ventricular function in patients receiving cardioprotective therapy compared to the placebo group [136,144].

In addition to neurohormonal therapies, other strategies such as dexrazoxane have also been explored to prevent anthracycline-induced cardiotoxicity. One such agent, dexrazoxane, has been investigated extensively in both pediatric and adult cancer patients for its ability to reduce the cardiovascular toxicity associated with anthracyclines [145]. Dexrazoxane is believed to exert its protective effects by chelating iron and preventing the formation of the topoisomerase IIβ–anthracycline complex, a key mechanism underlying cardiac damage [146,147]. Although it received approval from the U.S. Food and Drug Administration (FDA) in 1995 and is recommended by several cardiology societies, its use in clinical practice remains limited. The FDA specifically approved dexrazoxane for patients with advanced or metastatic breast cancer who have received a cumulative anthracycline dose of at least 300 mg/m^2^ [47]. A meta-analysis of nine studies with 2177 patients found that dexrazoxane significantly reduced the risk of clinical heart failure (RR: 0.19) and cardiac events (RR: 0.36), without influencing the oncological outcomes, overall survival, or progression-free survival. However, given the low quality of the evidence, further randomized trials are needed before it can be widely adopted in clinical practice [145].

Complementing the use of cardioprotective agents, modified chemotherapy formulations such as liposomal doxorubicin have also been developed to address anthracycline-associated cardiotoxicity [46]. This formulation involves encapsulating doxorubicin within lipid-based vesicles (liposomes), which modifies its pharmacokinetics and reduces its accumulation in non-target organs like the heart, while enhancing the delivery to tumor sites [148]. This innovative formulation has shown a more favorable toxicity profile compared to conventional anthracyclines. A meta-analysis of 19 clinical trials across both adjuvant and metastatic settings found that the use of liposomal doxorubicin significantly reduces the incidence of cardiotoxicity [149]. Due to its enhanced cardiac safety, liposomal doxorubicin is considered a more suitable option, particularly for patients at a higher risk for cardiac side effects.

## 10. Discussion and Future Directions

Breast cancer therapy using anthracyclines and trastuzumab has proven to be effective; however, cardiotoxicity remains a major challenge in clinical management. Cardiotoxicity represents a serious complication, as it may disrupt the treatment continuity and significantly reduce the QoL of breast cancer patients [76]. This review highlights that anthracycline-induced cardiotoxicity is dose-dependent and generally irreversible, while trastuzumab-related cardiotoxicity tends to be reversible, yet it remains a leading cause of therapy discontinuation [45].

The cardiotoxic mechanisms of doxorubicin and trastuzumab involve different pathways, yet both contribute to myocardial dysfunction. Doxorubicin induces oxidative stress, iron dysregulation, apoptosis, ferroptosis, and epigenetic alterations; whereas, trastuzumab inhibits HER2 signaling pathways and disrupts the mitochondrial integrity [47,85,86,99,100,101]. These findings are consistent with various studies demonstrating that cardiotoxicity is multifactorial and specific to the chemotherapeutic agent used.

An advanced age, hypertension, obesity, diabetes, and dyslipidemia are key risk factors that increase the likelihood of developing cardiotoxicity [65,118,120,124,125,126,127]. However, there are inconsistencies across studies in how these risk factors are defined and assessed, indicating the need for standardized risk stratification systems such as the HFA-ICOS [46].

Current diagnostic strategies, such as the measurement of GLS, allow for the earlier detection of cardiac dysfunction, although threshold values and clinical implementation still vary [45,47]. In addition, biomarkers such as troponin and natriuretic peptides show a promising predictive value but are not yet routinely used due to the need for further validation [46]. A baseline measurement of cardiac serum biomarkers is essential if changes in these biomarkers are to be used to detect a subclinical cardiac injury during cancer treatment [46]. The study also revealed that when combined with GLS, the troponin I measurement has a negative predictive value of 91% for the possibility of developing cardiotoxicity in the future [56]. The combined use of GLS and cardiac biomarkers may enhance the early detection of cardiotoxicity in patients receiving anti-HER2 therapies, especially in high-risk populations [45,55,150]. 

The research pinpointed the primary ways in which CTRCD diminishes the QoL for breast cancer patients. Interviews from 12 women with a history of breast cancer and CTRCD showed that those who received chemotherapy often experienced overwhelming fatigue, the mental burden of anxiety, a lack of understanding and acceptance, a lack of knowledge and acknowledgement, and a need for personalized care. The findings indicate that patients experience a heightened disease burden from cardiovascular complications, which adversely affects their physical, social, and psychosocial well-being. Furthermore, their healthcare encounters were often compromised by an insufficient acknowledgement of their concerns and suboptimal professional communication. In response, patients articulated a strong need for more personalized follow-up care. Implementing a tailored, multidisciplinary, and holistic care model holds the potential to substantially improve both patient healthcare experiences and the long-term QoL [151].

Consistent with the prior literature, a study conducted in Romania found a correlation between a decreased LVEF and patient-reported outcomes, such as increased fatigue and reduced physical ability—effects linked to the cardiotoxicity caused by treatment. Among patients with an LVEF below 50%, the average EORTC QLQ-C30 score was 63.17, while those with normal cardiac function (LVEF between 50 and 65%) had a slightly higher average score of 64.28, indicating a comparable quality of life, albeit with more variability. The study further showed that individuals with a lower LVEF (<50%) had somewhat reduced EORTC QLQ-C30 scores compared to those with normal heart function. These findings suggest that cardiotoxicity plays a meaningful role in the decline of the quality of life, even in patients with early-stage breast cancer [152].

While treatment with heart failure medication is linked to a functional improvement, a retrospective study from Japan indicates that the LVEF recovery is often incomplete. Specifically, the mean post-recovery LVEF of 56.9% remained significantly below the baseline value of 64.3%, implying a degree of persistent, irreversible myocardial damage or remodeling induced by anthracyclines. This is further complicated by the fact that the natural course of anthracycline-induced cardiomyopathy (AIC) is not fully understood, though the spontaneous recovery of the left ventricle systolic function has been previously reported. The study aligns with this, as a LVEF recovery without heart failure medication was observed in some patients, particularly those with a less severe initial dysfunction. Given that the mechanisms underlying such a spontaneous recovery are poorly characterized, identifying clinical parameters that can predict this outcome is crucial for effective patient stratification and a timely referral for specialized heart failure management [153]. According to a key observational study, patients who develop cardiotoxicity during treatment with trastuzumab typically exhibit a functional cardiac recovery upon the discontinuation of the therapy. Ewer (2005) reported that this improvement generally manifests within a few months, with an average recovery period of 1.5 months following the drug withdrawal [153,154].

Various cardioprotective therapies have been shown to be effective in preserving the left ventricular function and preventing a chemotherapy-induced cardiac injury. Agents such as lisinopril, enalapril, carvedilol, and candesartan have demonstrated the ability to reduce the risk of treatment discontinuation due to cardiac complications, both by improving cardiac function and preventing further structural damage [131,132,133]. Beyond neurohormonal therapies, dexrazoxane has also shown efficacy in preventing anthracycline-induced cardiotoxicity. However, its clinical use remains limited due to strict regulatory guidelines and concerns about potential oncologic effects; although to date, no strong evidence has supported these concerns [46,147,155,156].

In clinical practice, several challenges remain, such as the insufficient monitoring of the cumulative anthracycline dosage—which significantly increases the risk of cardiac complications—alongside the absence of routine cardiac function assessments and limited patient awareness regarding cardiotoxicity risks. Not all healthcare facilities have standardized protocols for monitoring cardiac function before, during, and after cancer treatment, leading to variability in clinical practices across centers. Some patients may be unable or unwilling to adhere to the recommended cardiac monitoring schedules due to financial barriers, low health literacy, or logistical issues such as limited access to transportation. Furthermore, collaboration between oncologists and cardiologists remains suboptimal, often resulting in delays in the detection and management of cardiotoxicity. To address these challenges, a comprehensive and systematic approach is needed, including routine cardiac monitoring, enhanced patient education regarding cardiotoxicity risks and prevention, and stronger interdisciplinary coordination among healthcare providers.

To achieve this, further research is required, including clinical trials to evaluate the effectiveness of various cardioprotective agents, such as ACE inhibitors, ARBs, beta-blockers, and dexrazoxane, in order to determine the most effective preventive therapies. Epidemiological studies and population-based data are also essential to better understand the incidence and specific risk factors for cardiotoxicity among different patient populations. Additionally, multidisciplinary collaboration models—such as cardio-oncology clinics and telemedicine—can improve patient access to cardiac monitoring. Through these various efforts, it is hoped that strategies for the prevention and management of cardiotoxicity can be optimized, allowing patients to continue receiving the full benefit of cancer therapy without an increased risk of cardiovascular complications.

## 11. Conclusion

Cardiotoxicity is a major challenge in breast cancer therapy, particularly with the use of anthracyclines and trastuzumab. Although their mechanisms differ, both agents can lead to cardiac dysfunction that affects treatment continuity. The early detection through LVEF and GLS assessments—as well as cardiac biomarkers, alongside the use of cardioprotective therapies such as ACE inhibitors, ARBs, beta-blockers, and dexrazoxane—has proven to be effective in preventing cardiac damage and preserving the left ventricular function.

However, clinical implementation remains limited due to the lack of standardized protocols and multidisciplinary collaboration. Therefore, an integrated approach is needed, including individualized risk stratification, routine cardiac monitoring, and the development of cardio-oncology services, supported by further research to optimize the prevention and management of cardiotoxicity and ensure that patients receive the full benefits of cancer therapy without increasing the risk of cardiac complications.

## Data Availability

Not applicable.

## Abbreviations

The following abbreviations are used in this manuscript:

Globocan	Global Burden of Cancer Study
LVEF	Left Ventricular Ejection Fraction
QoL	Quality of Life
WHO	World Health Organization
ASIR	Age-Standardized Incidence Rate
ER	Estrogen Receptor
PR	Progesterone Receptor
HER2	Human Epidermal Growth Factor Receptor 2
TNBC	Triple-Negative Breast Cancer
ASCO	American Society of Clinical Oncology
DFS	Disease-Free Survival
OS	Overall Survival
NOAH	Neoadjuvant Herceptin
EFS	Event-Free Survival
IDFS	Invasive Disease-Free Survival
T-DM1	Trastuzumab Emtansine
pCR	Pathologic Complete Response
ASE	American Society of Echocardiography
EACVI	European Society of Cardiovascular Imaging
ESC	European Society of Cardiology
CTRCD	Cancer Therapy-Related Cardiovascular Dysfunction
EF	Ejection Fraction
GLS	Global Longitudinal Strain
cTn	Cardiac Troponin
hs-cTn	High-Sensitivity Cardiac Troponin
TIC	Trastuzumab-Induced Cardiotoxicity
ROS	Reactive Oxygen Species
TOP2B	Topoisomerase II Beta
dsDNA	Double-Strand DNA
SQ-DOX	Semiquinone Doxorubicin
O_2_•^−^	Superoxide Anions
H_2_O_2_	Hydrogen Peroxide
SOD	Superoxide Dismutase
OH•	Hydroxyl Radicals
TRZ	Trastuzumab
NRG1	Neuregulin-1
NO	Nitric Oxide
MAPK	Mitogen-Activated Protein Kinase
PI3K	Phosphoinositide 3-Kinases
mPTP	Mitochondrial Permeability Transition Pores
HFA	Heart Failure Association
ICOS	International Cardio-Oncology Society
CRT-CVT	Cardiovascular Toxicity Associated with Cancer Treatment
ECG	Electrocardiogram
NPs	Natriuretic Peptides
ACEi	Angiotensin-Converting Enzyme Inhibitor
ARB	Angiotensin Receptor Blocker
RAAS	Renin–Angiotensin–Aldosterone System
FDA	Food and Drug Administration
AIC	Anthracycline-Induced Cardiomyopathy
